# Eugenol-Preconditioned Mesenchymal Stem Cell-Derived Extracellular Vesicles Promote Antioxidant Capacity of Tendon Stem Cells *In Vitro* and *In Vivo*

**DOI:** 10.1155/2022/3945195

**Published:** 2022-02-08

**Authors:** Xiangze Li, Zhan Su, Kaiying Shen, Qi Wang, Chencheng Xu, Fuqiang Wang, Yuchi Zhang, Dapeng Jiang

**Affiliations:** ^1^Department of General Surgery, Shanghai Children's Medical Center, Shanghai Jiao Tong University School of Medicine, Shanghai, China; ^2^Department of Orthopaedics, Heilongjiang Red Cross Sengong General Hospital, Heilongjiang, China; ^3^Department of Pediatric Surgery, Hongqi Hospital, Mudanjiang Medical University, Mudanjiang, China

## Abstract

Tendon stem cells (TSCs) are often exposed to oxidative stress at tendon injury sites, which impairs their physiological effect as well as therapeutic application. Recently, extracellular vesicles (EVs) derived from bone marrow mesenchymal stem cells (BMSCs) were shown to mediate cell protection and survival under stress conditions. The function of BMSC-EVs may be affected by pretreatment with various factors such as eugenol (EUG)—a powerful antioxidant. In our previous study, we found that H_2_O_2_ significantly impaired TSC proliferation and tenogenic differentiation capabilities. Apoptosis and intracellular ROS accumulation in TSCs were induced by H_2_O_2_. However, such H_2_O_2_-induced damage was prevented by treatment with EUG-BMSC-EVs. Furthermore, EUG-BMSC-EVs activated the Nrf2/HO-1 pathway to counteract H_2_O_2_-induced damage in TSCs. In a rat patellar tendon injury model, the ROS level was significantly higher than that in the normal tendon and TSCs not pretreated showed a poor therapeutic effect. However, EUG-BMSC-EV-pretreated TSCs significantly improved tenogenesis and matrix regeneration during tendon healing. Additionally, the EUG-BMSC-EV group had a significantly improved fiber arrangement. Overall, EUG-BMSC-EVs protected TSCs against oxidative stress and enhanced their functions in tendon injury. These findings provide a basis for potential clinical use of EUG-BMSC-EVs as a new therapeutic vehicle to facilitate TSC therapies for tendon regeneration.

## 1. Introduction

Tendons play important roles in transmitting forces from muscles to the skeleton. They are frequently injured by mechanical loading during both occupational and gymnastic activities [1]. In clinical medicine, improving the healing effectiveness after tendon injury remains a major challenge. The healing process of tendons is slow because of the inability of tendons to self-repair and the poor regenerative capability of tenocytes [2]. In recent years, tenocyte- and mesenchymal stromal cell-based therapeutic strategies have been applied to tendon injuries [3–5]. However, tenocytes and mesenchymal stromal cells may not be ideal cell sources for tendon repair because of their limited proliferative capability and the risk of ectopic bone formation [6]. Recently, tendon stem cells (TSCs) were shown to have potential for the regeneration of injured tendons [7–9]. However, nontenocyte differentiation and TSC dysfunction may occur after their transplantation because the local biochemical environment undergoes complicated changes after injuries, such as inflammation and oxidative stress [10, 11].

Previous studies have shown that oxidative stress diminishes the abilities of TSCs to self-renew, proliferate, and differentiate into tenocytes [12, 13]. TSC damage caused by oxidative stress is a major contributor to tendon degeneration and tendinopathy. It has also been reported that antagonizing posttraumatic oxidative stress with vitamin C reduces tendon adhesion [14]. Moreover, the proliferation and expression of tendon-related markers in TSCs are suppressed significantly by H_2_O_2_ treatment [10]. Consequently, it is necessary to find regulators that improve the function of TSCs under oxidative stress.

Extracellular vesicles released from bone marrow mesenchymal stem cells (BMSC-EVs) have recently been shown to mediate tissue regeneration as well as cell protection and survival under various pathophysiological conditions [15–17]. Recent studies have also demonstrated that MSC-EVs positively modulate the function of stem cells [18, 19]. However, BMSC-EVs have been found to play an anti-inflammatory role in tendon injury while their antioxidant capacity needs to be improved. As a consequence, BMSC-EV treatment does not achieve the goal of complete healing [20].

In recent years, cell preconditioning has attracted increasing attention. Preconditioning of cells with cytokines, hypoxic conditions, and small molecule compounds increases their capabilities [21–23]. Exosomes derived from pretreated MSCs also have therapeutic effects [24]. Eugenol (EUG) is a natural compound extracted from vegetable oil. It has a wide range of pharmacological effects that include antioxidant, anti-inflammatory, antibacterial, and antiviral effects [25]. Previous studies have demonstrated that EUG-pretreated adipogenic MSCs have enhanced antifibrosis abilities [26]. On the basis of the antioxidant effect of EUG, we speculated that preconditioning of BMSCs with EUG may improve the antioxidant effect of BMSC-EVs.

We hypothesized that EUG-BMSC-EVs may have a novel role in protecting TSCs against oxidative stress-induced damage. To test this hypothesis, EVs were isolated from BMSCs preconditioned with EUG. Then, the effects of EUG-BMSC-EVs on proliferation, apoptotic activity, tenocyte phenotype, and reactive oxygen species (ROS) accumulation in TSCs were investigated *in vitro*. The effects of TSCs pretreated with EUG-BMSC-EVs on mending tendon tissues were also characterized using a rat patellar tendon injury model.

## 2. Methods

### 2.1. Animals

Animal experiments were approved by the Animal Care and Use Committee, Shanghai Children's Medical Center, Shanghai Jiao Tong University School of Medicine (China). Male Sprague Dawley (SD) rats weighing 180-220 g at 8-10 weeks of age were provided by the Shanghai SLAC Laboratory Animal Company (No: SCMC-DWFLL-20200026).

### 2.2. Isolation and Culture of Rat BMSCs and TSCs

BMSCs were isolated from the bone marrow of SD rats as described previously [27]. Briefly, bone marrow was extracted from the femurs and tibias of rats and cultured in Dulbecco's modified Eagle's medium (Cat: C11885500BT, Gibco, USA) containing 10% fetal bovine serum (Cat: 10099141C, Gibco, USA) and 1% penicillin/streptomycin (Cat: 15140122, Gibco, USA). The medium was changed every 2 days. The BMSCs used in this study were between passages 3 and 5.

The isolation and culture methods of TSCs were previously established [28]. For primary cell cultures, patellar tendons were removed and cut into small sections; then, the fragments were digested with dispase (Cat: D4693, Sigma-Aldrich, USA) and type I collagenase (Cat: C0130, Sigma-Aldrich, USA) at 37°C for 1 h. The isolated cells were cultured in plates in Dulbecco's modified Eagle's medium containing 10% fetal bovine serum and 1% penicillin/streptomycin. TSCs formed colonies on the culture plates after 8–10 days. Cells from P2 to P3 after isolation were used in all experiments.

### 2.3. Preconditioning of BMSCs

EUG (Cat: E51791, Sigma-Aldrich, USA) was dissolved in ethanol. BMSCs were seeded in 96-well plates at a density of 5000 cells per well. Cells were rinsed with PBS after 24 h and preconditioned with EUG for another 24 h. After that, CCK8 assay was used to evaluate cell viability. 50 *μ*M EUG was chosen for our experiment.

For preconditioning, BMSCs were cultured in a complete medium until 70% confluent; then, they were washed twice with PBS. Serum-free medium was added for 24 h, followed by EUG-containing complete medium for 24 h. The BMSC group was added ethanol-containing complete medium.

### 2.4. Isolation and Identification of EVs

After preconditioning for 24 h, nonpreconditioned and EUG-preconditioned BMSCs were rinsed with PBS and cultured in a medium containing 10% exosome-depleted fetal bovine serum at 37°C for an additional 48 h. The culture medium was collected for obtaining EVs. The culture medium was centrifugated at 300 × g for 10 min and 2000 × g for 10 min to remove cellular debris. Then, the supernatant was centrifuged at 10,000 × g for 30 min. After that, cell-free supernatants were ultracentrifuged at 100,000 × g for 70 min twice. 50 *μ*L PBS was used to resuspend the EVs, and the resulting solution was used for downstream experiments. The total protein content in EVs was detected using a BCA protein assay kit (Cat: P0010, Beyotime, China). Western blotting, nanoparticle tracking analysis (NTA), and transmission electron microscopy (TEM) were used to identify the collected EVs. We also extracted protein from BMSCs for comparison.

### 2.5. Internalization of DiI PKH26-Labeled EVs into TSCs

BMSC-EVs and EUG-BMSC-EVs were labeled with DiI PKH26 (Cat: MINI26, Sigma-Aldrich, USA) as previously described [24]. EVs were washed in PBS to wash unbound DiI away. Then, TSCs were incubated with DiI-labeled EVs (10 *μ*g) for 12 h. TSCs were then fixed in 4% paraformaldehyde after being washed in PBS for three times and incubated with Hoechst 33342 (Cat: C1026, Beyotime, China) for 5 min at room temperature. Stained cells were observed under a laser confocal microscope.

### 2.6. H_2_O_2_ and EV Treatments

TSCs were subjected to 0.5 mM H_2_O_2_ for up to 24 h as described previously [29]. As soon as cells were exposed to H_2_O_2_, they were incubated in either control medium or medium containing BMSC-EVs or EUG-BMSC-EVs. To investigate whether the protective effect of EVs was associated with the activation of Nrf2/HO-1, TSCs were pretreated with ML385 (an Nrf2 inhibitor, Cat: HY-100523, Sigma-Aldrich, USA) for 4 h and cotreated with H_2_O_2_ for 24 h.

### 2.7. Cell Viability Assay

For cell viability assays, TSCs were plated in 96-well culture plates at a density of 5 × 10^3^ cells/well. After 24 h in culture, cells were treated with EVs and H_2_O_2_. Next, TSCs were cultured for another 24 h prior to assessing proliferation. The viability of TSCs was determined using the CCK8 assay.

### 2.8. Colony-Forming Assay

TSCs were seeded in culture dishes at 500 cells/well and incubated in DMEM supplemented with EVs and H_2_O_2_ for a further 7 days. The cells were stained with 0.5% Crystal Violet Staining Solution (Cat: C0121, Beyotime, China) for 10 min for counting the number of cell colonies. Colonies of less than 2 mm in diameter were ignored under a microscope.

### 2.9. Apoptosis Assay

TSC apoptosis was determined by annexin V-fluorescein isothiocyanate (FITC)/propidium iodide (PI) double staining. Harvested cells were suspended in 1x binding buffer (Cat: C1069M, Beyotime, China) and stained with FITC-conjugated annexin V and PI. Cells were analyzed by flow cytometry (FACSCanto™, BD, USA) within 1 h.

### 2.10. Detection of Intracellular ROS Accumulation

To assess intracellular peroxide accumulation, cells were stained with DCFH-DA (Cat: S0033M, Beyotime, China). Cells were analyzed under a fluorescence microscope. Fluorescence intensity was calculated for the quantitative analysis of ROS accumulation.

### 2.11. Animal Experiments

Generation of the rat patellar tendon injury model and the surgical procedures were performed as in previous studies [20]. The central one-third of the patellar tendon was removed to create a tendon injury. 12 rats were randomly divided into 2 groups (6 rats/group). In group II, the central one-third of the patellar tendon was removed to create a tendon injury and then we injected 30 *μ*L of fibrin sealant. However, rats in group I only got a skin incision at the same site. At 2 days after surgery, rats were sacrificed. The normal tendon in group I and the injured tendon in group II was collected and processed via cryosectioning for further analysis. DCFH-DA was used to detect ROS produced in a tendon.

64 rats underwent surgery for partial resection of the patellar tendon followed by treatment immediately. Rats were randomly divided into 4 groups (16 rats/group). In the control group, the defect was filled with 30 *μ*L volume of fibrin sealant alone. In the TSC group, 30 *μ*L volume of fibrin with 5 × 10^4^ TSCs (without BMSC-EV treatment) was injected into the tendon injury. In the BMSC-EV+TSC group, 30 *μ*L volume of fibrin with 5 × 10^4^ TSCs (pretreated with 60 *μ*g/mL BMSC-EVs for 24 h) was inoculated into the tendon injury. In the EUG-BMSC-EV+TSC group, 30 *μ*L volume of fibrin with 5 × 10^4^ TSCs (pretreated with 60 *μ*g/mL EUG-BMSC-EVs for 24 h) was inoculated into the tendon injury. At 1 and 2 weeks after surgery, 8 rats in each group were sacrificed, and the injured patellar tendons were harvested for histology, immunohistochemistry, protein, and mRNA analysis.

### 2.12. Histology and Immunohistochemistry

At weeks 1 and 2 after treatments, collected rat patellar tendon tissues were fixed in 4% paraformaldehyde (Cat: G1101, Servicebio, China) for 24 h. Specimens were used for immunohistochemistry to examine the expression of proliferating cell nuclear antigen (PCNA, Cat: 10205-2-AP, dilution: 1 : 500, Proteintech, USA), tenascin C (TNC, Cat: 67710-1-Ig, dilution: 1 : 500, Proteintech, USA), tenomodulin (TNMD, Cat: ab203676, dilution: 1 : 400, Abcam, UK), scleraxis (SCXA, Cat: DF13293, dilution: 1 : 400, Affinity, USA), collagen type I (COLI, Cat: 66761-1-Ig, dilution: 1 : 500, Proteintech, USA), and collagen type III (COLIII, Cat: 22734-1-AP, dilution: 1 : 500, Proteintech, USA). The specimens were embedded in paraffin. Sections of 4 *μ*m thickness were cut and deparaffinized in xylene followed by hydration, and then, they were placed in 3% hydrogen peroxide to block endogenous activity. Blocking of the sections was performed with 5% bovine serum albumin for 20 min. After that, sections were incubated overnight at 4°C with primary antibodies. Slides were then incubated with a secondary antibody in the dark at 37°C for 30 min. Images were observed with a digital pathology slide scanner (KF-PRO-120, KFBIO).

The specimens were also used for hematoxylin-eosin (H&E) or Masson trichrome staining. Histopathological analysis of the stained rat patellar tendon tissue was performed using the fiber alignment score as previously described: 0 = 0% to 25% parallel fiber alignment; 1 = 25% to 50% parallel fiber alignment; 2 = 50% to 75% parallel fiber alignment; and 3 = 75% to 100% parallel fiber alignment [14].

### 2.13. Reverse Transcription-Quantitative Polymerase Chain Reaction (RT-qPCR)

Gene expression in cells and healing tendons was determined via real-time PCR. Total RNA was extracted with Trizol reagent (Cat: 15596018, Invitrogen, USA). cDNA was synthesized using a PrimeScript™ RT Master Mix Kit (Cat: RR036A, TAKARA, China). RT-PCR was carried out with the CFX96 Real-Time PCR Detection System (Bio-Rad, USA). Total RNA isolation, cDNA synthesis, and gene expression assays were performed as in previous studies [29]. Glyceraldehyde-3-phosphate dehydrogenase (GAPDH) was used as an endogenous reference gene. Relative gene expression levels were analyzed with the 2^*ΔΔ*CT^ formula and then normalized to controls.

Rat-specific primers used for TNMD, SCX, TNC, Cat, PCNA, Sod1, Nfe2l2, HO-1, collagen type I, collagen type III, bFGF, and GAPDH are shown in Table 1.

### 2.14. Western Blotting Analysis

RIPA lysis buffer (Cat: C1053, Applygen, China) containing Cocktail (50x) (Cat: P1265, Applygen, China) was used to prepare tissues and cells. After isolating proteins from homogenates, immunoblotting was performed overnight at 4°C using the following rabbit primary antibodies: Col III (Cat: 22734-1-AP, dilution: 1 : 1000, Proteintech, USA), Col I (Cat: 66761-1-Ig, dilution: 1 : 1000, Proteintech, USA), CD9 (Cat: ab263019, dilution: 1 : 1000,Abcam, UK), CD63 (Cat: ab134045, dilution: 1 : 1000, Abcam, UK), TSG101 (Cat: ab133586, dilution: 1 : 1000, Abcam, UK), TNC (Cat: 67710-1-Ig, dilution: 1 : 1000, Proteintech, USA), TNMD (Cat: ab203676, dilution: 1 : 1000, Abcam, UK), SCXA (Cat: DF13293, dilution: 1 : 1000, Affinity, USA), PCNA (Cat: 10205-2-AP, dilution: 1 : 1000, Proteintech, USA), poly ADP-ribose polymerase 1 (PARP1, Cat: 13371-1-AP, dilution: 1 : 1000, Proteintech, USA), catalase (Cat: 21260-1-AP, dilution: 1 : 1000, Proteintech, USA), and GAPDH (Cat: 60004-1-Ig, dilution: 1 : 1000, Proteintech, USA). Horseradish peroxidase- (HRP-) conjugated secondary antibodies (Cat: 15015&15014, dilution: 1 : 10000, Proteintech, China) were then incubated with the membranes for 1 h at room temperature. Chemiluminescent signals were developed with an enhanced chemiluminescence (Millipore, USA) and detected by the ChemiDoc imaging system (Tanon, China).

### 2.15. Statistical Analysis

Experiments were carried out more than three times *in vitro* and *in vivo*. All data were expressed as means ± standard deviation from at least three separate experiments. We used Student's *t*-test to compare differences between two groups. In order to compare differences in more than two groups, analysis of variance followed by Tukey's multiple comparison test was used. *P* < 0.05 was considered statistically significant. The analysis was all performed in GraphPad Prism (GraphPad Software Inc., USA).

## 3. Results

### 3.1. Characterization and Internalization of BMSC-EVs

**Figure 1:**
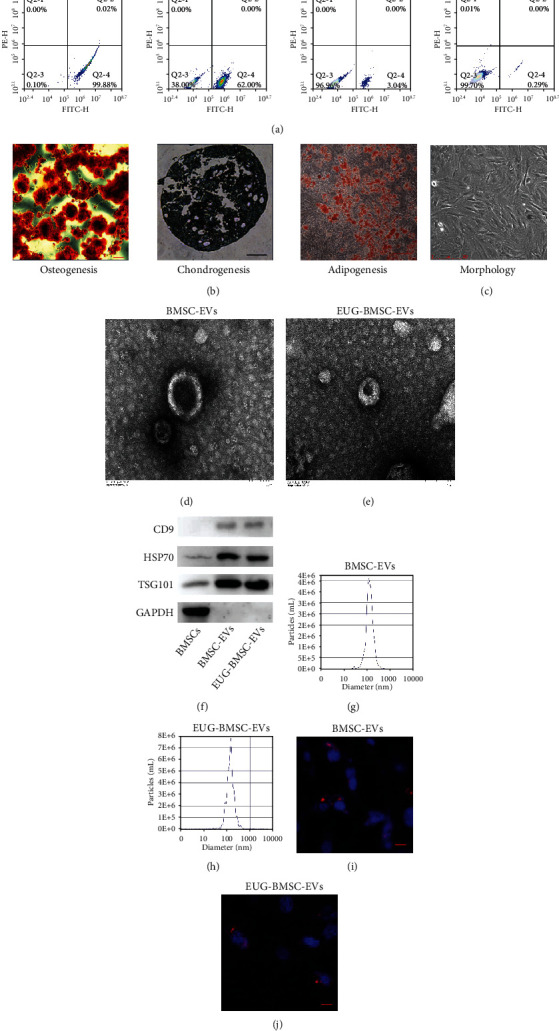
Characterization of BMSCs and EVs. (a) FACS analysis for detection of BMSC surface markers. (b) The osteogenic, chondrogenic, and adipogenic differentiation potentials of BMSCs. Scale bar: 200 *μ*m. (c) Morphology of BMSCs. Scale bar: 250 *μ*m. (d, e) Morphology using transmission electron microscopy. (f) Western blot was used to detect the markers of EVs. (g, h) NTA was used to examine the particle size distribution. (i, j) Internalization of EVs by TSCs. EVs were labeled with DiI PKH26 while the nuclei of TSCs were labeled with Hoechst 33342. Scale bar: 20 *μ*m.

BMSCs were positive for cell surface markers, such as CD90 (99.88%) and CD44 (62.00%), but negative for the hematopoietic markers CD34 (3.04%) and C11b (0.29%) (Figure 1(a)). We also analyzed the morphology and multipotency of BMSCs (Figures 1(b) and 1(c)). We characterized BMSC-EVs and EUG-BMSC-EVs through TEM, NTA analysis, and western blotting. TEM analysis showed that the shape of EVs was round or spherical (Figures 1(d) and 1(e)). Western blotting analysis revealed that the expression levels of CD9, HSP70, and TSG101 were significantly higher in EVs compared with BMSCs (Figure 1(f)). Moreover, as shown by nanoparticle tracking analysis (NTA), EVs were uniform in size with a diameter of approximately 30-200 nm; the average diameter of EVs was 130 nm (Figures 1(g) and 1(h)).

The internalization of EVs was observed under a laser confocal microscope. The red fluorescence of EVs was localized in the cytoplasm of TSCs, indicating that EVs were internalized by TSCs (Figures 1(i) and 1(j)).

### 3.2. EUG-BMSC-EVs Enhanced the Viability and Proliferation of H_2_O_2_-Treated TSCs

**Figure 2:**
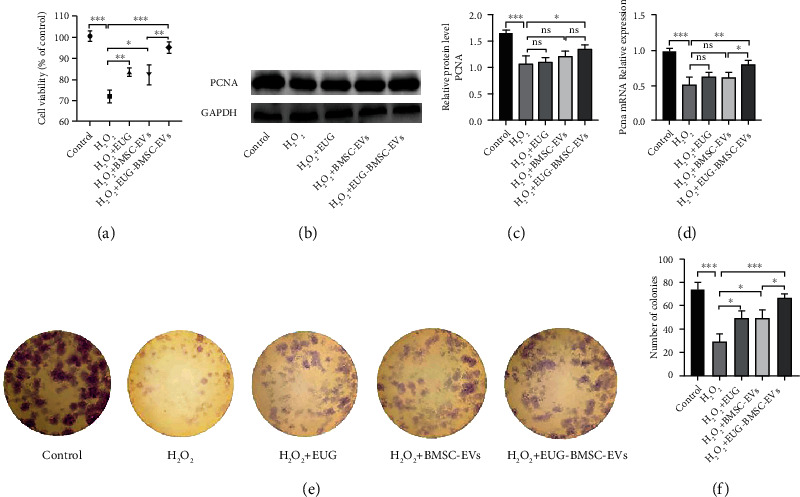
EUG-BMSC-EVs enhanced the viability and proliferation of H_2_O_2_-treated TSCs. (a) CCK8 assay was performed to assess TSC viability. (b, c) The protein and mRNA expression of PCNA in TSCs. (d) The expression of PCNA gene. (e, f) Colony-forming assay was performed to assess proliferation capacity of TSCs. ^∗^*P* < 0.05, ^∗∗^*P* < 0.01, and ^∗∗∗^*P* < 0.001.

We first examined the effects of H_2_O_2_ treatment on the viability of TSCs. As shown in Figure 2(a), H_2_O_2_ at a concentration of 0.5 mM for 24 h significantly impaired the viability of TSCs compared with controls. TSCs exhibited enhanced viability after treatment with BMSC-EVs, and the EUG-BMSC-EVs group showed better viability.

H_2_O_2_ at a concentration of 0.5 mM for 24 h significantly impaired the expression of PCNA and the colony formation capacity of TSCs. Nonpreconditioned BMSC-EVs can hardly increase the PCNA level. In contrast, EUG-BMSC-EVs significantly increased the PCNA expression and enhanced the relative number and average size of colonies (Figures 2(b)–2(f)).

### 3.3. Effect of EUG-BMSC-EVs on the Tenogenic Differentiation of H_2_O_2_-Treated TSCs

**Figure 3:**
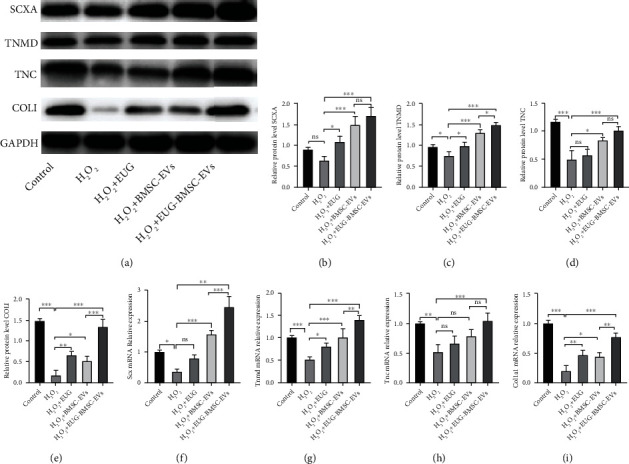
EUG-BMSC-EVs protected tenogenic differentiation of H_2_O_2_-treated TSCs. (a–e) Western blot was used to examine the expression of SCXA, TNMD, TNC, and COLI. (f–i) The expression of tenocyte-related genes, SCX, TNMD, TNC, and Col1a1. ^∗^*P* < 0.05, ^∗∗^*P* < 0.01, and ^∗∗∗^*P* < 0.001.

We examined the level of COLI, TNC (a glycoprotein of the extracellular matrix), TNMD (tenogenesis-related marker), and SCXA (tenogenic transcription factor), which are related to tenogenic differentiation [30]. Their expression levels were decreased by H_2_O_2_ treatment, but EUG-BMSC-EVs reversed these effects (Figures 3(a)–3(i)). TSCs subjected to EUG-BMSC-EVs exhibited a significant increase in the expression of these markers compared with the group treated with H_2_O_2_. Although BMSC-EVs can also protect TSCs against H_2_O_2_, their effect was inferior to EUG-BMSC-EVs.

### 3.4. Effect of EUG-BMSC-EVs on H_2_O_2_-Induced TSC Apoptosis

**Figure 4:**
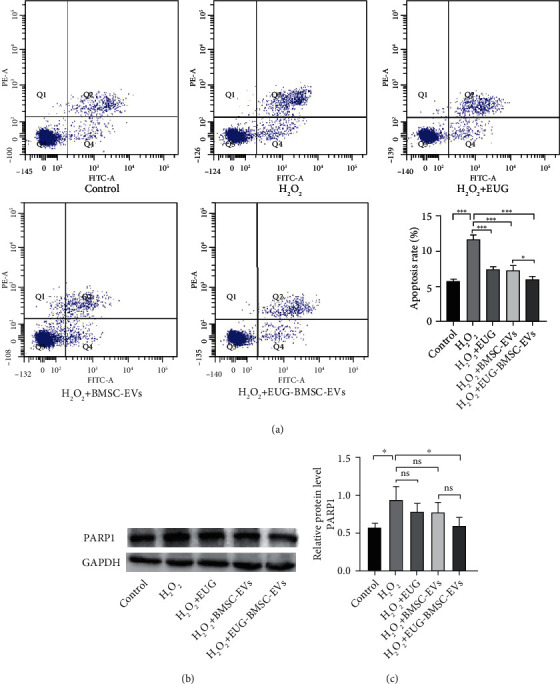
EUG-BMSC-EVs decreased H_2_O_2_-induced apoptosis. (a) Flow cytometry was used to assess the apoptosis of the TSCs. (b, c) Western blot was used to examine the expression of PARP1. ^∗^*P* < 0.05 and ^∗∗∗^*P* < 0.001.

Apoptosis rates were gauged by flow cytometry analysis, which showed that H_2_O_2_ induced the apoptosis of TSCs after culture for 24 h *in vitro*. The proportion of apoptotic cells was 12% ± 1% after treatment with H_2_O_2_. We next investigated the effects of EUG-BMSC-EVs on H_2_O_2_-induced apoptosis in TSCs. Treatment of TSCs with EUG and EVs decreased the percentage of apoptotic cells and the expression of PARP1, and EUG-BMSC-EVs were more effective than BMSC-EVs or EUG alone (Figures 4(a)–4(c)).

### 3.5. Effect of EUG-BMSC-EVs on Antioxidant Capacity of H_2_O_2_-Treated TSCs

**Figure 5:**
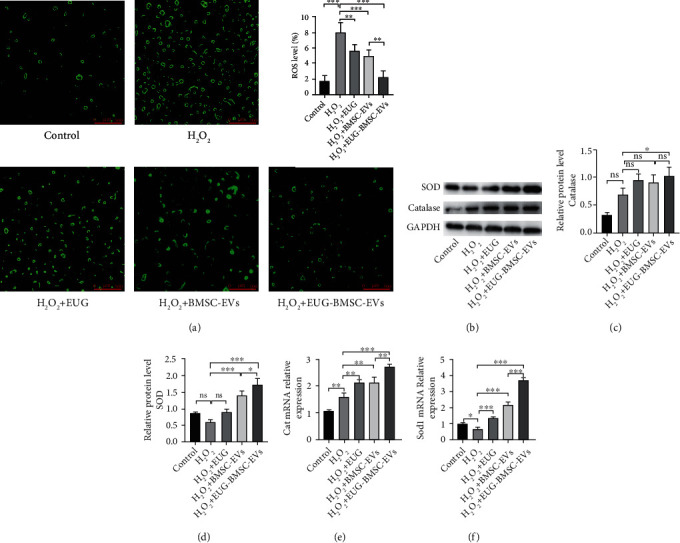
EUG-BMSC-EVs enhanced antioxidant capacity of H_2_O_2_-treated TSCs. (a) Detection of intracellular ROS accumulation by immunofluorescence assay. (b–d) Western blot was used to detect the expression of catalase and SOD. (e, f) The expression of Cat and Sod1 genes. Bars: 500 *μ*m. Data are represented as mean ± SD. ^∗^*P* < 0.05, ^∗∗^*P* < 0.01, and ^∗∗∗^*P* < 0.001.

Exposure to H_2_O_2_ leads to increased intracellular ROS generation. To examine the mechanisms underlying the protective effects of EUG-BMSC-EVs on TSCs, we also detected intracellular ROS level. The intensity of ROS fluorescence in cells was suppressed by treatment with EUG-BMSC-EVs (Figure 5(a)). We also detected the expression of SOD and catalase in H_2_O_2_-treated TSCs. As we predicted, EUG and BMSC-EVs increased the levels of catalase and SOD while both indexes were significantly higher in the EUG-BMSC-EVs group (Figures 5(b)–5(f)).

### 3.6. Nrf2/HO-1 Signaling Mediates the Protective Effects of EUG-BMSC-EVs on H_2_O_2_-Induced TSCs

**Figure 6:**
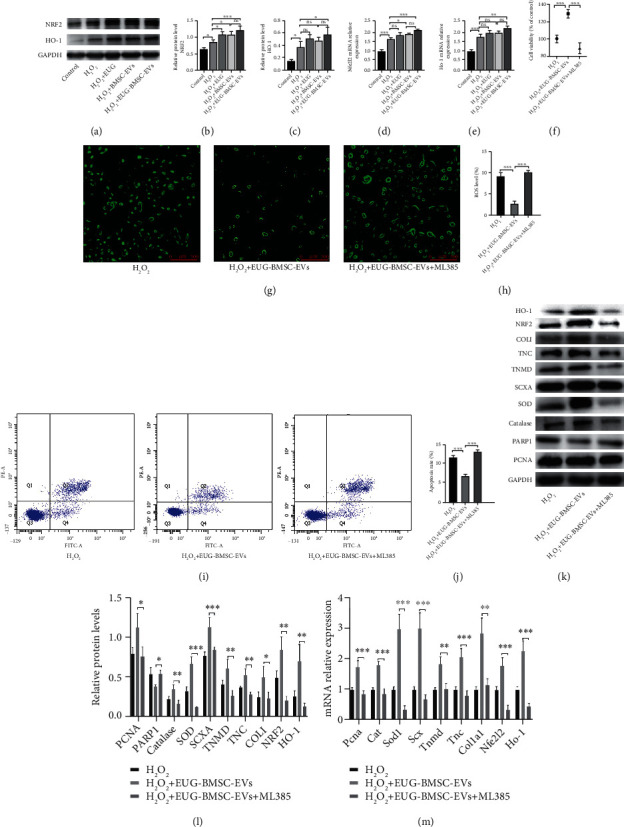
EUG-BMSC-EVs protected TSCs against H_2_O_2_ via Nrf2/HO-1 signaling pathway. (a–c) Western blot was used to examine the expression of NRF2 and HO-1 in H_2_O_2_-treated TSCs. (d, e) The expression of Nfe2l2 and HO-1 genes. (f) CCK8 assay was performed to assess TSC viability after ML385 treatment. (g, h) Apoptosis of the TSCs after ML385 treatment was detected by flow cytometry. (i, j) The intracellular ROS accumulation was detected by immunofluorescence assay after ML385 treatment. (k, l) Western blot was used to examine the expression of PCNA, catalase, SOD, PARP1, SCXA, TNMD, TNC, COLI, NRF2, and HO-1 after ML385 treatment. (m) The expression of PCNA, Cat, Sod1, SCX, TNMD, TNC, Col1a1, Nfe2l2, and HO-1 genes after ML385 treatment. Bars: 500 *μ*m. Data are represented as mean ± SD. ^∗^*P* < 0.05, ^∗∗^*P* < 0.01, and ^∗∗∗^*P* < 0.001.

Western blotting was performed to detect activation of the Nrf2/HO-1 signaling pathway by EUG-BMSC-EVs in this study. EUG-BMSC-EVs significantly augmented Nrf2 and HO-1 expression levels in H_2_O_2_-induced TSCs compared with H_2_O_2_ only groups (Figures 6(a)–6(e)). To explore whether the cytoprotective effects of EUG-BMSC-EVs were dependent on Nrf2 activation, we pretreated TSCs with ML385 (an inhibitor of Nrf2). The result showed that ML385 dramatically attenuated the effect of EUG-BMSC-EVs on antiapoptosis and antioxidant in H_2_O_2_-treated TSCs. Moreover, inhibition of Nrf2 activation blocked the protective effects of EUG-BMSC-EVs on the impaired cell proliferation and tenogenesis of H_2_O_2_-induced TSCs (Figures 6(f)–6(m)).

### 3.7. Effect of EUG-BMSC-EVs Pretreated TSCs on Tendon Morphology

**Figure 7:**
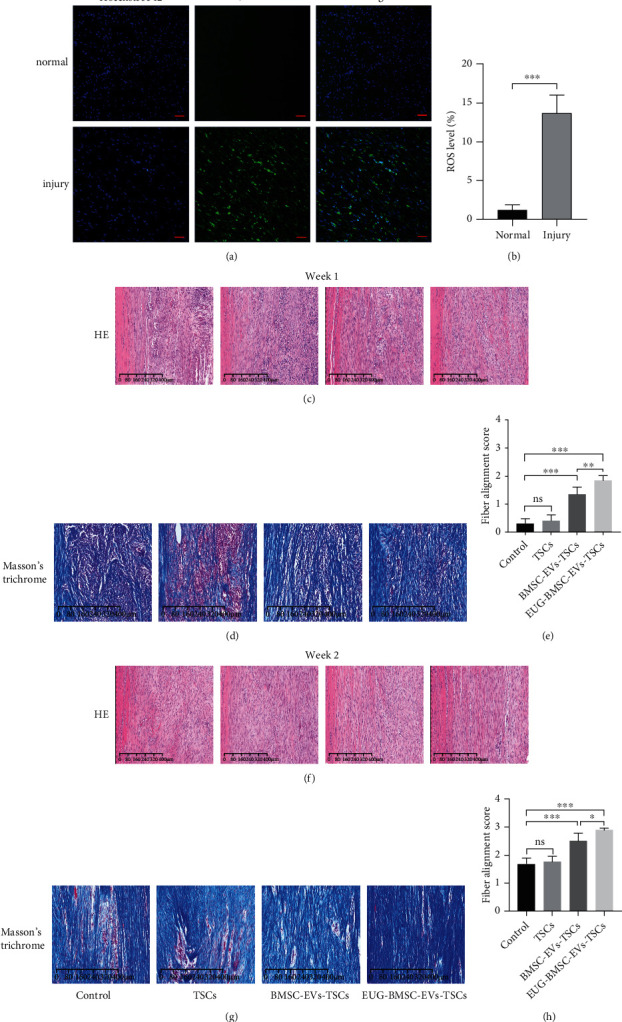
EUG-BMSC-EVs improved the healing of tendon injury. (a, b) ROS levels in normal and injured tendon. Bars: 200 *μ*m. (c) The HE staining of tendon injury on the patellar tendon at 1 week. (d) Masson's trichrome staining of tendon injury at 1 week after surgery. (e) Fiber alignment score of the tendon injury rats (*n* = 8 donors). (f) The HE staining of tendon injury on the patellar tendon at 2 weeks. (g) Masson's trichrome staining of tendon injury at 2 weeks after surgery. (h) Fiber alignment score of the tendon injury rats (*n* = 8 donors). Bars: 400 *μ*m. Data are represented as mean ± SD. ^∗^*P* < 0.05, ^∗∗^*P* < 0.01, and ^∗∗∗^*P* < 0.001.

We examine the ROS levels in a normal and injured tendon 2 days after surgery. The expression of ROS in an injured tendon was significantly higher than the normal tendon (Figures 7(a) and 7(b)). This phenomenon indicated intense oxidative stress at tendon injury sites.

We investigated the effects of TSCs pretreated with EUG-BMSC-EVs on tendon regeneration by H&E and Masson's trichrome staining. We used fiber alignment score to evaluate the tendon: 0 = 0% to 25% parallel fiber alignment; 1 = 25% to 50% parallel fiber alignment; 2 = 50% to 75% parallel fiber alignment; and 3 = 75% to 100% parallel fiber alignment. At 1 week after implantation, organized and compact collagen fibers were observed in the EUG-BMSC-EV-pretreated TSCs implantation groups (Figures 7(c) and 7(d)). Compared with the control groups, the EUG-BMSC-EV-treated group showed significantly improved fiber arrangement. The fiber alignment score was significantly higher for the group treated with BMSC-EV-TSC compared with the control group and the TSC group (Figure 7(e)). As we predicted, the EUG-BMSC-EVs promoted TSC functions better when compared with the BMSC-EV group. After 2 weeks, we analyze the fiber alignment score of each group. The results showed that the EUG-BMSC-EV-treated group almost achieved perfect healing (Figures 7(f)–7(h)).

### 3.8. EUG-BMSC-EVs Enhanced Proliferation and Tenogenesis of TSCs during Tendon Healing

**Figure 8:**
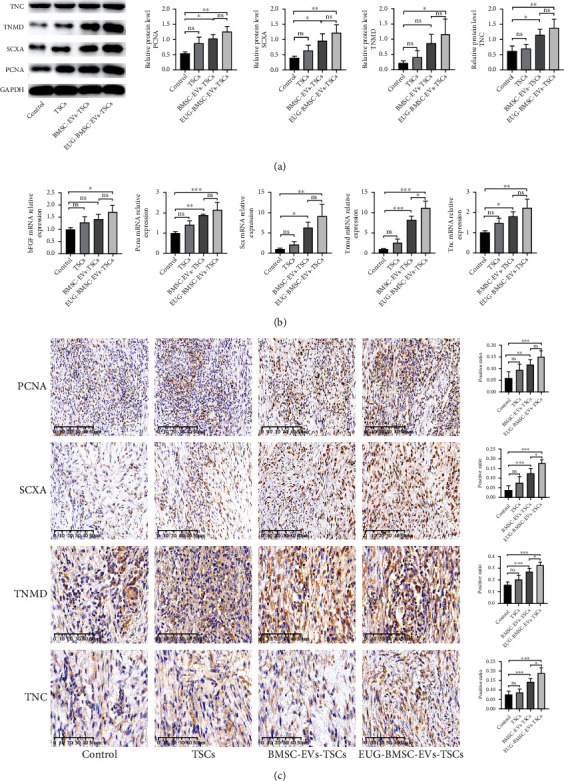
EUG-BMSC-EV-pretreated TSCs promoted proliferation and tenogenesis at week 1. (a) Western blot was used to examine the expression of PCNA, SCXA, TNMD, and TNC in tendon injury. (b) The gene expression of bFGF, PCNA, SCX, TNMD, and TNC in tendon injury. (c) Immunohistochemistry assay was performed to assess the expression of PCNA, SCXA, TNMD, and TNC in tendon injury. Bars (PCNA, SCXA): 100 *μ*m; bars (TNMD, TNC): 50 *μ*m. Data are represented as mean ± SD. ^∗^*P* < 0.05, ^∗∗^*P* < 0.01, and ^∗∗∗^*P* < 0.001.

The impact of TSCs on cell growth and differentiation during tendon healing was assessed in this study. At 1 week after injury, the expression of PCNA was higher in the EUG-BMSC-EV group. The expression of SCXA and TNMD was significantly elevated in the EUG-BMSC-EV-treated group compared with other groups. In addition, TNC was also enhanced in the EUG-BMSC-EV group. We also found that EVs derived from EUG-preconditioned BMSCs can significantly enhance TSC capability in tendon injury compared with BMSC-EVs. Basic fibroblast growth factor (bFGF) was reported to promote tendon repair. Compared with tendon tissues from other groups, repaired tendons from the TSC group (treated with EUG-BMSC-EVs) exhibited a higher level of bFGF (Figures 8(a) and 8(b)).

**Figure 9:**
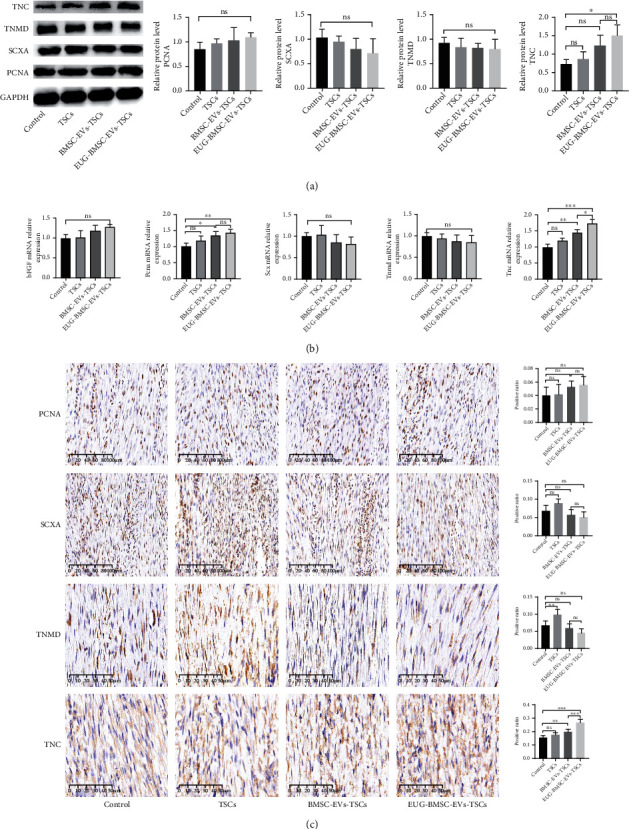
Effects of EUG-BMSC-EV-pretreated TSCs on proliferation and tenogenesis at week 2. (a) Western blot was used to examine the expression of PCNA, SCXA, TNMD, and TNC in tendon injury. (b) The gene expression of bFGF, PCNA, SCX, TNMD, and TNC in tendon injury. (c) Immunohistochemistry assay was performed to assess the expression of PCNA, SCXA, TNMD, and TNC in tendon injury. Bars (PCNA, SCXA): 100 *μ*m; bars (TNMD, TNC): 50 *μ*m. Data are represented as mean ± SD. ^∗^*P* < 0.05, ^∗∗^*P* < 0.01, and ^∗∗∗^*P* < 0.001.

Immunohistochemical staining showed that PCNA-expressing cells were found in the center of the window defect at week 1. High expression of SCXA, TNMD, and TNC was observed in the EUG-BMSC-EV-treated group compared with other groups (Figure 8(c)). At 2 weeks after injury, the significant enhancement of PCNA, SCXA, TNMD, and bFGF had decreased while TNC remain higher in the EUG-BMSC-EV-treated group (Figures 9(a)–9(c)). We speculate that this phenomenon may correlate with better repair in the EUG-BMSC-EV group.

### 3.9. Effect of EUG-BMSC-EV-Pretreated TSCs on Matrix Regeneration during Tendon Healing

**Figure 10:**
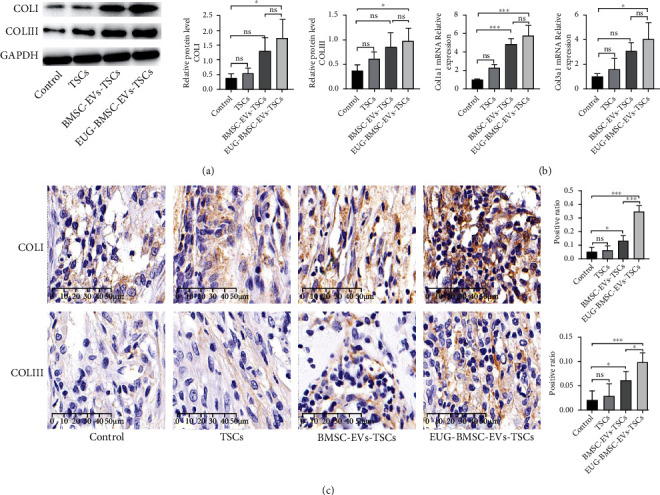
EUG-BMSC-EV-pretreated TSCs enhanced matrix regeneration during tendon healing at week 1. (a) Western blot was used to examine the expression of COLI and COLIII in tendon injury. (b) The expression of Col1a1 and Col3a1 genes in tendon injury. (c) The expression of COLI and COLIII was detected by immunohistochemistry assay in tendon injury. Bars: 50 *μ*m. Data are represented as mean ± SD. ^∗^*P* < 0.05 and ^∗∗∗^*P* < 0.001.

**Figure 11:**
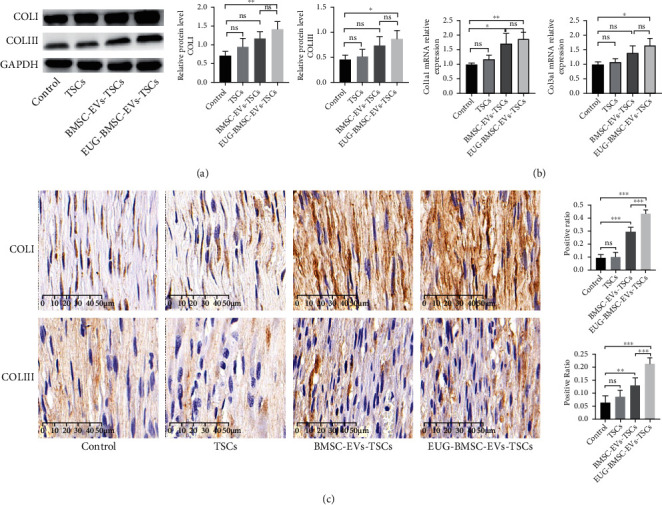
Effect of EUG-BMSC-EV-pretreated TSCs on matrix regeneration during tendon healing at week 2. (a) Western blot was used to examine the expression of COLI and COLIII in tendon injury. (b) The expression of Col1a1 and Col3a1 genes in tendon injury. (c) The expression of COLI and COLIII was detected by immunohistochemistry assay in tendon injury. Bars: 50 *μ*m. Data are represented as mean ± SD. ^∗^*P* < 0.05, ^∗∗^*P* < 0.01, and ^∗∗∗^*P* < 0.001.

COLI was increased in healing tendons from the TSC group (treated with BMSC-EVs) compared with control tendons. The addition of BMSC-EV-treated TSCs to injured tendons also enhanced the COLIII expression level in repaired tendons (Figures 10(a) and 10(b)). When BMSC was preconditioned with EVs, EVs derived from them significantly enhanced the TSC capability which was reflected by COLI and COLIII levels. These results support the potential of EUG-BMSC-EV-treated TSCs to regulate tendon matrix formation. Immunohistochemical staining also showed higher expression of both collagen in the EUG-BMSC-EV-treated TSC group than in other groups (Figure 10(c)). After 2 weeks, we can find excellent collagen accumulation in the EUG-BMSC-EV group. The expression of COLI was also higher than COLIII, which revealed high quality of tendon healing (Figures 11(a)–11(c)).

## 4. Discussion

TSCs implanted into tendon injury sites are exposed to various cellular stresses that impair their self-renewal, proliferation, and differentiation capabilities [12, 13, 31]. Oxidative stress in tendon injury sites has been identified as a major factor that contributes to degenerative events and extracellular matrix organization in tendons [32, 33]. Moreover, oxidative stress-induced damage of TSCs provides a potential therapeutic target for tendon repair [10, 12]. Previous studies have indicated that conditioned medium from MSCs alleviates oxidative stress of expanded umbilical cord blood cells [34]. Recent studies have demonstrated that BMSCs mediate wound healing through secretion of EVs [35–37]. MSC-EVs also suppress liver injury development via antioxidant activities [38]. BMSC-EVs contain various prosurvival factors that reduce apoptosis, inflammation, and oxidative stress during tissue repair and degenerative diseases. Recent studies have also demonstrated the importance of MSC-EVs in regulating the properties of cells during tissue regeneration [16, 39]. On the basis of these findings, we determined whether BMSC-EVs modulated the impaired function of TSCs under oxidative stress.

In recent years, attention has been focused on the preconditioning of MSCs to enhance their functions. Salidroside reduces the production of ROS in endothelial cells induced by hyperglycemia, thereby regulating apoptosis induced by oxidative stress to play a cellular protective role. Moreover, preconditioning with salidroside enhances the repair function of MSCs in diabetic patients [40]. Preconditioning MSCs with eicosapentaenoic acid also demonstrated superior effects on inflammation as well as tissue remodeling compared to nonpreconditioned MSCs in both models of allergic asthma and sepsis [41, 42]. Hydroxycamptothecin (HCPT), a DNA topoisomerase I inhibitor, inhibits excessive proliferation and induces fibroblast apoptosis. It has been reported that EVs from human bone marrow mesenchymal stem cells pretreated with HCPT have a strong therapeutic effect on tendon adhesion after tendon injury [43]. EVs derived from LPS-preconditioned MSCs better regulate polarization of macrophages and inhibit chronic inflammation [44]. Therefore, we speculated that preconditioned MSCs would have a better therapeutic effect under treatment with certain substances. EUG is a natural anti-inflammatory agent and antioxidant [45]. We hypothesized that EVs derived from EUG-preconditioned BMSCs would have a better antioxidant capacity and would better ameliorate impairment of TSCs induced by oxidative stress than BMSC-EVs alone.

Cell proliferation and viability are important for tenocyte regeneration. The proliferation of TSCs was decreased by H_2_O_2_, which might account for the poor repair in tendons under oxidative stress. Moreover, we found that H_2_O_2_ significantly impaired the viability of TSCs. These results support that oxidative stress is detrimental to TSCs for tendon healing.

We also investigated the effects of H_2_O_2_ on the tenogenic differentiation potential. TSCs in the H_2_O_2_-treated group expressed significantly lower levels of COLI, TNC, TNMD, and SCX compared with the control group. TNMD is important for early tenogenic differentiation, and SCX positively regulates the expression of TNMD and COLI [46, 47]. TNC is a glycoprotein of the extracellular matrix, which is fundamental for cell-cell and cell-matrix interactions [48]. Our results showed that oxidative stress impaired the tenogenic differentiation ability of TSCs, which may be a challenge in TSC-based therapy [10]. However, TSCs pretreated with EUG-BMSC-EVs retained their ability, such as proliferation, viability, and tenogenic differentiation.

Previous reports indicate that oxidative stress is a potential proapoptotic factor in stem cells [49–51]. We found that TSCs exposed to H_2_O_2_ exhibited a significant increase in apoptosis. Increased apoptotic activity in tendon tissue often correlates with an altered tendon tissue composition and damaged matrix during repair. A previous study has shown that BMSC-EVs modulate several biological processes that include cell apoptosis [52]. We speculated that BMSC-EVs may also affect H_2_O_2_-induced apoptosis of TSCs. PARP1 plays an important role in DNA damage repair and is used as an indicator of cell apoptosis. We used PARP1 to reflect TSCs apoptosis [53]. In this study, BMSC-EVs relieved apoptosis of TSCs exposed to oxidative stress. These data also indicated that EUG-BMSC-EVs were superior to BMSC-EVs in protecting TSCs from H_2_O_2_-induced damage.

Intracellular oxidative stress decreases collagen expression in tissues, which leads to degeneration [54]. We found that H_2_O_2_-treated TSCs exhibited an increase in ROS accumulation. ROS are essential modulators that control physiological activities during tissue repair, such as cell growth, proliferation, migration, differentiation, and apoptosis. Production of ROS is involved in the pathological mechanism of tendon degeneration. Catalase, an important antioxidant enzyme in scavenging ROS, plays an important role in maintaining the balance of redox state. In our study, catalase level increased after H_2_O_2_ treatment, which suggested increased oxidative stress and the involvement of ROS. The oxidative stress-induced activation of the Nrf2/HO-1 pathway results in increased expression of downstream antioxidant enzymes including catalase. The phenomenon was in accordance with a previous study [55]. Considering that BMSC-EVs often function as antioxidants, we detected ROS and catalase levels in TSCs under oxidative stress. As we predicted, treatment with EUG-BMSC-EVs significantly decreased intracellular ROS levels and increased catalase levels in H_2_O_2_-treated TSCs. These results suggested that EUG-BMSC-EVs protected TSCs from oxidative stress.

Nrf2 plays a critical role in preventing cell and tissue damage caused by oxidative stress. It controls the transcription of several cytoprotective genes in response to oxidative stress [56, 57]. Previous studies have revealed that oxidative stress contributes to tendon degeneration and Nrf2/HO-1 upregulation increases the capability of TSCs to cope with H_2_O_2_-induced damage [13]. Our results were consistent with previous findings and showed that cell pretreatment with ML385 (an inhibitor of Nrf2) attenuated EUG-BMSC-EV-induced cytoprotective effects. EUG-BMSC-EVs potentially protected against oxidative-related tendon physiological dysfunctions by activating the Nrf2/HO-1 pathway.

Our results also showed that transplantation of TSCs pretreated with EUG-BMSC-EVs promoted tendon repair at 14 days after injury. This was evidenced by the improved fiber alignment score after the transplantation of pretreated TSCs. However, the group implanted with TSCs alone did not show obvious effects. In previous studies, some people found that TSCs were effective, while others found that TSCs alone had little effect [58, 59]. We found that the TSC group had a better effect than the control group while there was no significant difference in many indexes. We inferred that this result may be associated with the survival of TSCs. Untreated TSCs exhibit limited survival in wound tissue and cause unsatisfactory repair [10]. Pretreatment with EUG-BMSC-EVs enhanced the capability of TSCs to adapt to pathophysiological conditions. The improved tissue quality with EUG-BMSC-EV-treated TSCs was further supported by Masson's trichrome staining results. In particular, more regenerated collagen fibers were observed in the EUG-BMSC-EV-treated TSC group.

In this study, EUG-BMSC-EVs promoted tenogenic differentiation and increased the expression of tendon-related genes and proteins in the healing tendon. Collagen is the major protein in tendons and provides an environment to maintain a stretch for tendon cells [60]. In the patellar tendon injury model, implantation of EUG-BMSC-EV-treated TSCs significantly enhanced COLI and COLIII production.

We also examined the expression of tenocyte-related genes. As expected, the expression of SCX was elevated by EUG-BMSC-EV-pretreated TSCs in healing tendons at 1 week in our study. TNMD is involved in tendon maturation [30], and it was significantly increased in the EUG-BMSC-EV-pretreated TSC implantation group. bFGF is involved in fibroblast/tenocyte growth, and its expression was increased in healing tendon tissues following implantation of EUG-BMSC-EV-pretreated TSCs. These findings suggest that EUG-BMSC-EVs maintained the spontaneous tenogenic differentiation and growth potential of TSCs in healing tissues. We also found that these indexes were decreased after 2 weeks. We believe this phenomenon may be correlated with the better repair in the EUG-BMSC-EV group. During the early stage of repair, TSCs pretreated with EUG-BMSC-EVs actively participated in the reconstruction of tendons and accelerated wound tissue recovery. Because this also resulted in rapid consumption of TSCs, the repair process slowed after 2 weeks.

The current study has some limitations. First, we did not explore how EUG enhances the antioxidant capacity of BMSC-EVs. Therefore, the components in EVs altered by EUG remain to be explored. Second, we did not monitor the survival of TSCs in the injured tendons of our animal experiments, and only wound healing and the expression of related proteins in the tendons were used to assess repair. The survival and function of cells at the tendon also remain to be explored. We will do further research to explore the mechanism and make EUG-BMSC-EVs justifiable for clinical therapy.

## 5. Conclusions

In brief, we demonstrate that EUG-BMSC-EVs could protect TSCs from damage induced by oxidative stress through activating the Nrf2/HO-1 pathway. Implantation of EUG-BMSC-EV-pretreated TSCs is a viable approach to improve the quality of tendon healing. These findings provide us with a novel therapeutic strategy for tendon repair in the future.

## Figures and Tables

**Table 1:** Rat-specific primers used for qRT-PCR analysis.

TNMD	Forward	5′-CCAGACAAGCAAGCGAGGAAGAC-3′
	Reverse	5′-ACAGACCCTGCGGCAGTAGC-3′
SCX	Forward	5′-CAACGTGCTACTGGTGGGTGAAG-3′
	Reverse	5′-TGTTCTCGCCGCCGTCTCTG-3′
TNC	Forward	5′-AAAGCAGCCACCCGCTATTA-3′
	Reverse	5′-TCAGGTTCTTTGGCTGTGGAG-3′
PCNA	Forward	5′-CGGCGTGAACCTACAGAGCATG-3′
	Reverse	5′-GCAGCGGTATGTGTCGAAGCC-3′
Sod1	Forward	5′-TGGCGGTCCAGCGGATGAAG-3′
	Reverse	5′-CGGCCAATGATGGAATGCTCTCC-3′
Cat	Forward	5′-GCGAATGGAGAGGCAGTGTACTG-3′
	Reverse	5′-GGTCTTCCTGTGCAAGTCTTCCTG-3′
Nfe2l2	Forward	5′-GCCTTCCTCTGCTGCCATTAGTC-3′
	Reverse	5′-TGCCTTCAGTGTGCTTCTGGTTG-3′
HO-1	Forward	5′-CAGACAGAGTTTCTTCGCCAGAGG-3′
	Reverse	5′-TGTGAGGACCCATCGCAGGAG-3′
Col1a1	Forward	5′-AGAAAGGATCTCCTGGTGC-3′
	Reverse	5′-ACGTTCACCACTTGCTCCA-3′
Col3a1	Forward	5′-TGCACCTGGCAAAAACGG-3′
	Reverse	5′-TTCCATTTTCTCCTGGAGG-3′
bFGF	Forward	5′-GACGATGACGATGATGATGACTCCTC-3′
	Reverse	5′-GTAACGAACCTTGTAGCCTCCGATC-3′
GAPDH	Forward	5′-TGACTCTACCCACGGCAAGTTCAA-3′
	Reverse	5′-ACGACATACTCAGCACCAGCATCA-3′

## Data Availability

All data generated or analyzed during this study are included in this manuscript.

## Abbreviations

BCA: Bicinchoninic acid
BMSC: Bone marrow mesenchymal stem cells
Cat: Catalogue
COL: Collagen
DMEM: Dulbecco's modified Eagle's medium
EUG: Eugenol
FBS: Fetal bovine serum
GAPDH: Glyceraldehyde-3-phosphate dehydrogenase
H&E: Hematoxylin-eosin
HO-1: Heme oxygenase 1
MSCs: Mesenchymal stem cells
NTA: Nanoparticle tracking analysis
Nrf2/Nfe2l2: Nuclear factor, erythroid 2-like 2
PARP1: Poly ADP-ribose polymerase 1
PBS: Phosphate-buffered saline
PCNA: Proliferating cell nuclear antigen
ROS: Reactive oxygen species
SCX: Scleraxis
SD: Standard deviation
TEM: Transmission electron microscopy
TNMD: Tenomodulin
TNC: Tenascin C
TSCs: Tendon stem/progenitor cells

## References

[1] Pillai D. S., Dhinsa B. S., Khan W. S. (2017). Tissue engineering in Achilles tendon reconstruction; the role of stem cells, growth factors and scaffolds. *Current Stem Cell Research & Therapy*.

[2] Morita W., Dakin S. G., Snelling S. J. B., Carr A. J. (2017). Cytokines in tendon disease. *Bone & Joint Research*.

[3] Turlo A. J., Ashraf Kharaz Y., Clegg P. D., Anderson J., Peffers M. J. (2018). Donor age affects proteome composition of tenocyte-derived engineered tendon. *BMC Biotechnology*.

[4] Shen H., Jayaram R., Yoneda S. (2018). The effect of adipose-derived stem cell sheets and CTGF on early flexor tendon healing in a canine model. *Scientific Reports*.

[5] Zhou Y., Zhang J., Wu H., Hogan M. V., Wang J. H. C. (2015). The differential effects of leukocyte-containing and pure platelet-rich plasma (PRP) on tendon stem/progenitor cells - implications of PRP application for the clinical treatment of tendon injuries. *Stem Cell Research & Therapy*.

[6] Blum B., Bar-Nur O., Golan-Lev T., Benvenisty N. (2009). The anti-apoptotic gene survivin contributes to teratoma formation by human embryonic stem cells. *Nature Biotechnology*.

[7] Zhang C., Zhang E., Yang L. (2018). Histone deacetylase inhibitor treated cell sheet from mouse tendon stem/progenitor cells promotes tendon repair. *Biomaterials*.

[8] Tarafder S., Chen E., Jun Y. (2017). Tendon stem/progenitor cells regulate inflammation in tendon healingviaJNK and STAT3 signaling. *FASEB Journal*.

[9] Chen J., Zhang E., Zhang W. (2017). FosPromotes early stage teno-lineage differentiation of tendon stem/progenitor cells in tendon. *Stem Cells Translational Medicine*.

[10] Lee Y. W., Fu S. C., Yeung M. Y., Lau C. M., Chan K. M., Hung L. K. (2017). Effects of redox modulation on cell proliferation, viability, and migration in cultured rat and human tendon progenitor cells. *Oxidative Medicine and Cellular Longevity*.

[11] Tang C., Chen Y., Huang J. (2018). The roles of inflammatory mediators and immunocytes in tendinopathy. *Journal of Orthopaedic Translation*.

[12] Chen H., Ge H. A., Wu G. B., Cheng B., Lu Y., Jiang C. (2016). Autophagy prevents oxidative stress-induced loss of self-renewal capacity and stemness in human tendon stem cells by reducing ROS accumulation. *Cellular Physiology and Biochemistry*.

[13] Sun W., Meng J., Wang Z. (2017). Proanthocyanidins attenuation of HO-induced oxidative damage in tendon-derived stem cells via upregulating Nrf-2 signaling pathway. *BioMed Research International*.

[14] Hung L. K., Fu S. C., Lee Y. W., Mok T. Y., Chan K. M. (2013). Local vitamin-C injection reduced tendon adhesion in a chicken model of flexor digitorum profundus tendon injury. *Journal of Bone and Joint Surgery*.

[15] Liu W., Wang Y., Gong F. (2019). Exosomes derived from bone mesenchymal stem cells repair traumatic spinal cord injury by suppressing the activation of A1 neurotoxic reactive astrocytes. *Journal of Neurotrauma*.

[16] Abreu S. C., Lopes-Pacheco M., Weiss D. J., Rocco P. R. M. (2021). Mesenchymal stromal cell-derived extracellular vesicles in lung diseases: current status and perspectives. *Frontiers in Cell and Developmental Biology*.

[17] Yang J., Liu X. X., Fan H. (2015). Extracellular vesicles derived from bone marrow mesenchymal stem cells protect against experimental colitis via attenuating colon inflammation, oxidative stress and apoptosis. *PLoS One*.

[18] Firoozi S., Pahlavan S., Ghanian M. H. (2020). Mesenchymal stem cell-derived extracellular vesicles alone or in conjunction with a SDKP-conjugated self-assembling peptide improve a rat model of myocardial infarction. *Biochemical and Biophysical Research Communications*.

[19] Bai Y., Han Y. D., Yan X. L. (2018). Adipose mesenchymal stem cell-derived exosomes stimulated by hydrogen peroxide enhanced skin flap recovery in ischemia-reperfusion injury. *Biochemical and Biophysical Research Communications*.

[20] Shi Z., Wang Q., Jiang D. (2019). Extracellular vesicles from bone marrow-derived multipotent mesenchymal stromal cells regulate inflammation and enhance tendon healing. *Journal of Translational Medicine*.

[21] Bai M., Zhang L., Fu B. (2018). IL-17A improves the efficacy of mesenchymal stem cells in ischemic-reperfusion renal injury by increasing Treg percentages by the COX-2/PGE2 pathway. *Kidney International*.

[22] Liu Y., Ren H., Zhou Y. (2019). The hypoxia conditioned mesenchymal stem cells promote hepatocellular carcinoma progression through YAP mediated lipogenesis reprogramming. *Journal of Experimental & Clinical Cancer Research*.

[23] Yang Z., He C., He J., Chu J., Liu H., Deng X. (2018). Curcumin-mediated bone marrow mesenchymal stem cell sheets create a favorable immune microenvironment for adult full-thickness cutaneous wound healing. *Stem Cell Research & Therapy*.

[24] Hu Y., Tao R., Chen L. (2021). Exosomes derived from pioglitazone-pretreated MSCs accelerate diabetic wound healing through enhancing angiogenesis. *Journal of Nanobiotechnology*.

[25] Ulanowska M., Olas B. (2021). Biological properties and prospects for the application of eugenol-a review. *International Journal of Molecular Sciences*.

[26] Fathy M., Okabe M., Saad Eldien H. M., Yoshida T. (2020). AT-MSCs antifibrotic activity is improved by eugenol through modulation of TGF-*β*/Smad signaling pathway in rats. *Molecules*.

[27] Wang L. L., Yin X. F., Chu X. C., Zhang Y. B., Gong X. N. (2018). Retracted: Platelet-derived growth factor subunit B is required for tendon-bone healing using bone marrow-derived mesenchymal stem cells after rotator cuff repair in rats. *Journal of Cellular Biochemistry*.

[28] Bi Y., Ehirchiou D., Kilts T. M. (2007). Identification of tendon stem/progenitor cells and the role of the extracellular matrix in their niche. *Nature Medicine*.

[29] Sun Y., Chen H., Ye H. (2020). Nudt21-mediated alternative polyadenylation of HMGA2 3′-UTR impairs stemness of human tendon stem cell. *Aging*.

[30] Lin D., Alberton P., Caceres M. D., Volkmer E., Schieker M., Docheva D. (2017). Tenomodulin is essential for prevention of adipocyte accumulation and fibrovascular scar formation during early tendon healing. *Cell Death & Disease*.

[31] Jiang D., Xu B., Yang M., Zhao Z., Zhang Y., Li Z. (2014). Efficacy of tendon stem cells in fibroblast-derived matrix for tendon tissue engineering. *Cytotherapy*.

[32] DePhillipo N. N., Aman Z. S., Kennedy M. I., Begley J. P., Moatshe G., LaPrade R. F. (2018). Efficacy of vitamin C supplementation on collagen synthesis and oxidative stress after musculoskeletal injuries: a systematic review. *Orthopaedic Journal of Sports Medicine*.

[33] Kim R. J., Hah Y. S., Sung C. M., Kang J. R., Park H. B. (2014). Do antioxidants inhibit oxidative-stress-induced autophagy of tenofibroblasts?. *Journal of Orthopaedic Research*.

[34] Kadekar D., Rangole S., Kale V., Limaye L. (2016). Conditioned medium from placental mesenchymal stem cells reduces oxidative stress during the cryopreservation of *ex vivo* expanded umbilical cord blood cells. *PLoS One*.

[35] Wu P., Zhang B., Shi H., Qian H., Xu W. (2018). MSC-exosome: a novel cell-free therapy for cutaneous regeneration. *Cytotherapy*.

[36] Shi Q., Qian Z., Liu D. (2017). GMSC-derived exosomes combined with a chitosan/silk hydrogel sponge accelerates wound healing in a diabetic rat skin defect model. *Frontiers in Physiology*.

[37] Kim Y. J., Yoo S. M., Park H. H. (2017). Exosomes derived from human umbilical cord blood mesenchymal stem cells stimulates rejuvenation of human skin. *Biochemical and Biophysical Research Communications*.

[38] Jiang W., Tan Y., Cai M. (2018). Human umbilical cord MSC-derived exosomes suppress the development of CCl-induced liver injury through antioxidant effect. *Stem Cells International*.

[39] Zhang S., Chuah S. J., Lai R. C., Hui J. H. P., Lim S. K., Toh W. S. (2018). MSC exosomes mediate cartilage repair by enhancing proliferation, attenuating apoptosis and modulating immune reactivity. *Biomaterials*.

[40] Ariyanti A. D., Zhang J., Marcelina O. (2019). Salidroside-pretreated mesenchymal stem cells enhance diabetic wound healing by promoting paracrine function and survival of mesenchymal stem cells under hyperglycemia. *Stem Cells Translational Medicine*.

[41] Silva J. D., Lopes-Pacheco M., de Castro L. L. (2019). Eicosapentaenoic acid potentiates the therapeutic effects of adipose tissue-derived mesenchymal stromal cells on lung and distal organ injury in experimental sepsis. *Stem Cell Research & Therapy*.

[42] Abreu S. C., Lopes-Pacheco M., da Silva A. L. (2018). Eicosapentaenoic acid enhances the effects of mesenchymal stromal cell therapy in experimental allergic asthma. *Frontiers Immunolgy*.

[43] Li J., Yao Z., Xiong H. (2020). Extracellular vesicles from hydroxycamptothecin primed umbilical cord stem cells enhance anti-adhesion potential for treatment of tendon injury. *Stem Cell Research & Therapy*.

[44] Ti D., Hao H., Tong C. (2015). LPS-preconditioned mesenchymal stromal cells modify macrophage polarization for resolution of chronic inflammation via exosome-shuttled let-7b. *Journal of Translational Medicine*.

[45] Barboza J. N., Filho C. S. M. B., Silva R. O., Medeiros J. V. R., Sousa D. P. (2018). An overview on the anti-inflammatory potential and antioxidant profile of eugenol. *Oxidative Medicine and Cellular Longevity*.

[46] Schweitzer R., Chyung J. H., Murtaugh L. C. (2001). Analysis of the tendon cell fate using scleraxis, a specific marker for tendons and ligaments. *Development*.

[47] Dex S., Alberton P., Willkomm L. (2017). Tenomodulin is required for tendon endurance running and collagen I fibril adaptation to mechanical load. *EBioMedicine*.

[48] Roth S. P., Schubert S., Scheibe P., Groß C., Brehm W., Burk J. (2018). Growth factor-mediated tenogenic induction of multipotent mesenchymal stromal cells is altered by the microenvironment of tendon matrix. *Cell Transplantation*.

[49] Cao H., Zhi Y., Xu H., Fang H., Jia X. (2019). Zearalenone causes embryotoxicity and induces oxidative stress and apoptosis in differentiated human embryonic stem cells. *Toxicology in Vitro*.

[50] Shin S., Choi J. W., Lim S. (2018). Anti-apoptotic effects of adipose-derived adherent stromal cells in mesenchymal stem cells exposed to oxidative stress. *Cell Biochemistry and Function*.

[51] Tian J., Gu L., Adams A., Wang X., Huang R. (2018). Pellino-1 protects periodontal ligament stem cells against H2O2-Induced apoptosis via activation of NF-*κ*B signaling. *Molecular Biotechnology*.

[52] Cheng X., Zhang G., Zhang L. (2018). Mesenchymal stem cells deliver exogenous miR-21viaexosomes to inhibit nucleus pulposus cell apoptosis and reduce intervertebral disc degeneration. *Journal of Cellular and Molecular Medicine*.

[53] Ray Chaudhuri A., Nussenzweig A. (2017). The multifaceted roles of PARP1 in DNA repair and chromatin remodelling. *Nature Reviews Molecular Cell Biology*.

[54] Galicka A., Krętowski R., Nazaruk J., Cechowska-Pasko M. (2014). Anethole prevents hydrogen peroxide-induced apoptosis and collagen metabolism alterations in human skin fibroblasts. *Molecular and Cellular Biochemistry*.

[55] Prakash R., Fauzia E., Siddiqui A. J. (2021). Oxidative stress enhances autophagy-mediated death of stem cells through Erk1/2 signaling pathway - implications for neurotransplantations. *Stem Cell Reviews and Reports*.

[56] Huang B., Liu J., Fu S. (2020). *α*-Cyperone attenuates H2O2-Induced oxidative stress and apoptosis in SH-SY5Y cells via activation of Nrf2. *Frontiers in Pharmacology*.

[57] Aladaileh S. H., Hussein O. E., Abukhalil M. H. (2019). Formononetin upregulates Nrf2/HO-1 signaling and prevents oxidative stress, inflammation, and kidney injury in methotrexate-induced rats. *Antioxidants*.

[58] Komatsu I., Wang J. H. C., Iwasaki K., Shimizu T., Okano T. (2016). The effect of tendon stem/progenitor cell (TSC) sheet on the early tendon healing in a rat Achilles tendon injury model. *Acta Biomaterialia*.

[59] Chen L., Liu J. P., Tang K. L. (2014). Tendon derived stem cells promote platelet-rich plasma healing in collagenase-induced rat Achilles tendinopathy. *Cellular Physiology and Biochemistry*.

[60] Gaut L., Duprez D. (2016). Tendon development and diseases. *Wiley Interdisciplinary Reviews Developmental Biology*.

